# Supplementary material for: The Relative Dose Intensity Changes during Cycles of Standard Regimens in Patients with Diffuse Large B-Cell Lymphoma
Source: Cancers (Basel). 2023 Sep 7;15(18):4458. doi: 10.3390/cancers15184458 (PMC10526837; doi:10.3390/cancers15184458)
Supplement: Supplementary file 1 [file cancers-15-04458-s001.zip › cancers-2465941-supplementary.pdf]

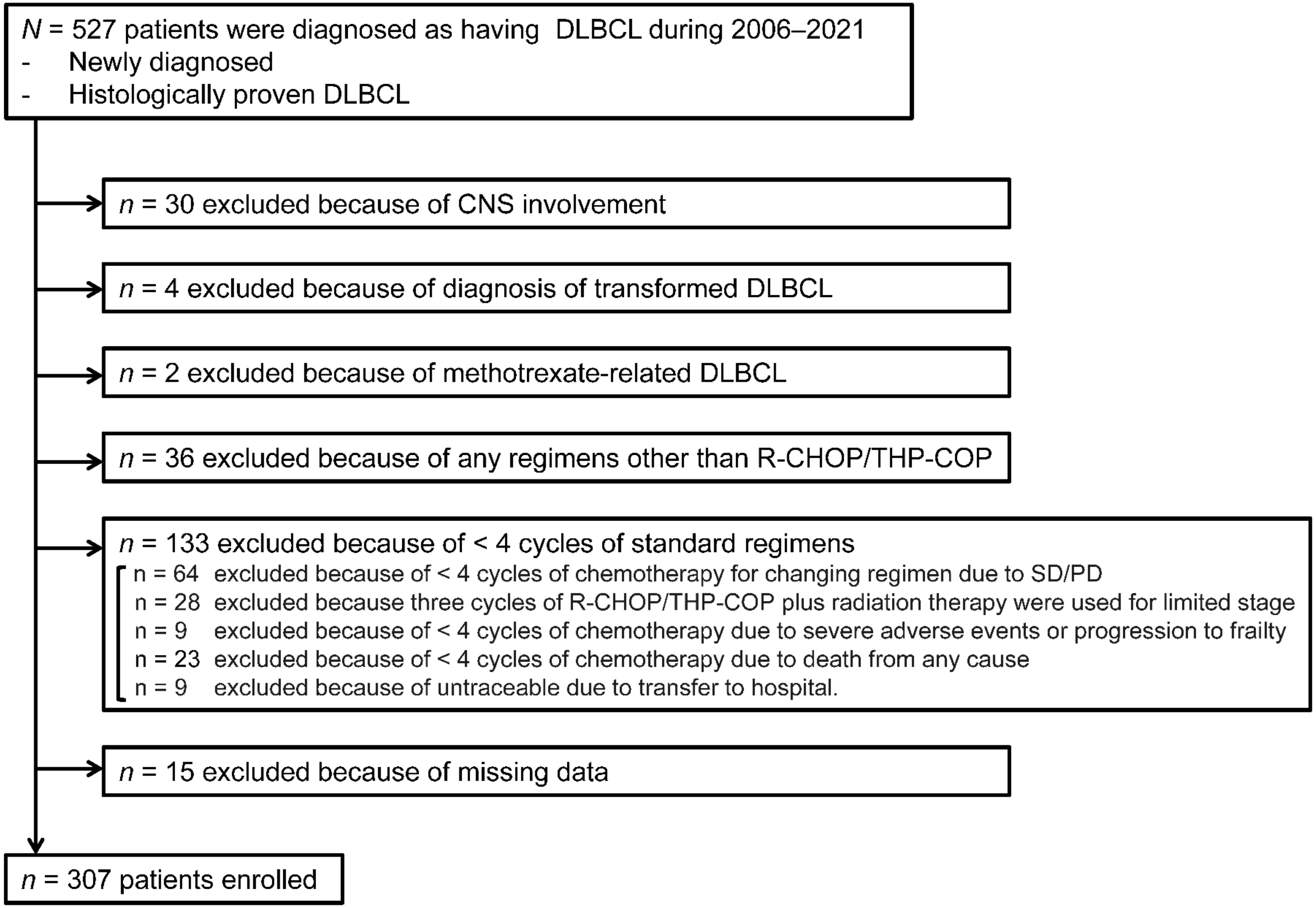

CNS = central nervous system; DLBCL = diffuse large B-cell lymphoma.

Supplementary Figure S1. Flow chart of patient selection.

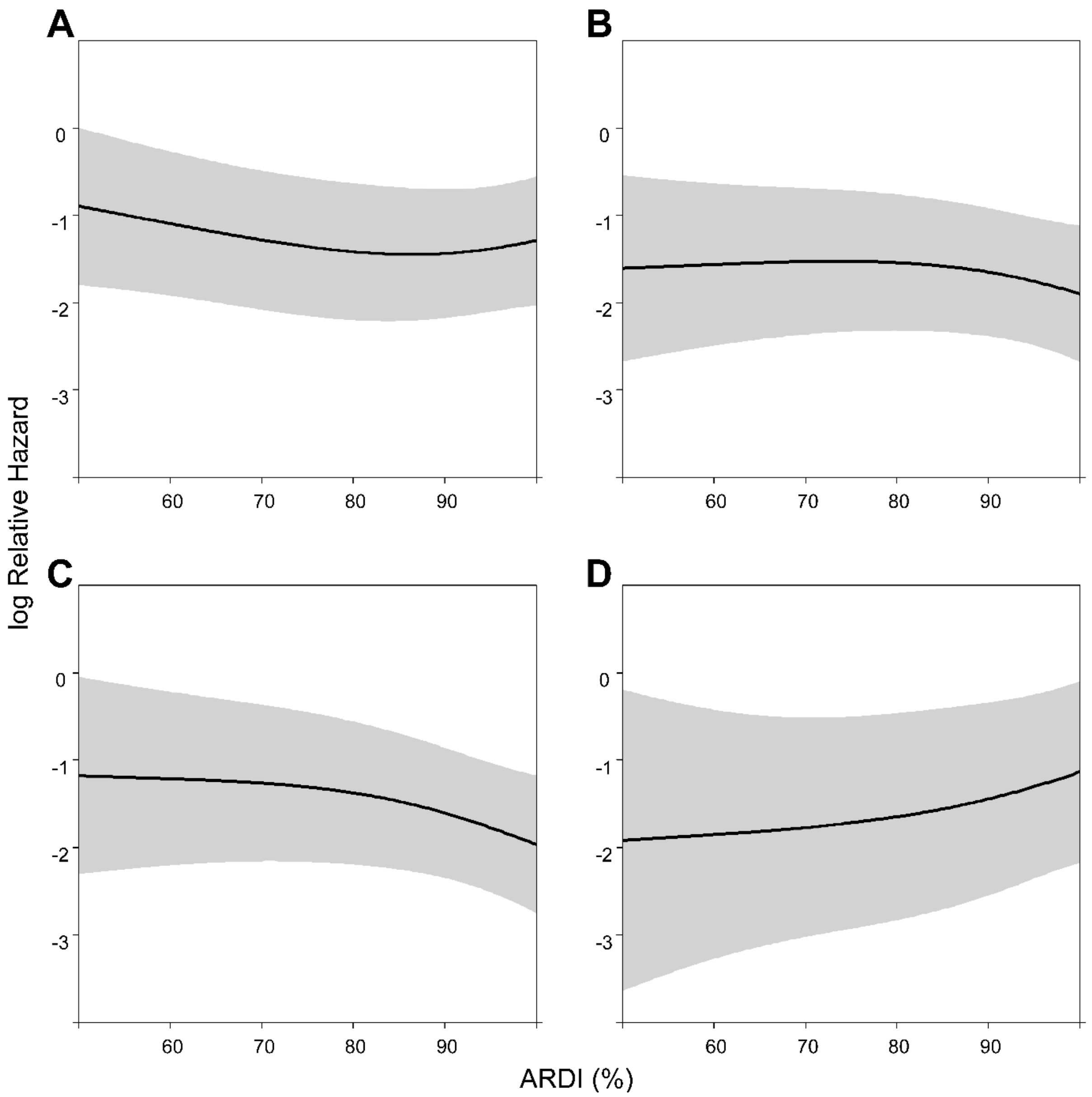

Supplementary Figure S2. Association between average relative dose intensity and all-cause mortality risk using a multivariate Cox hazards model with restricted cubic spline with 3 knots. (**A**) Up to the second cycle. (**B**) Up to the fourth cycle. (**C**) Up to the sixth cycle. (**D)** Up to the eighth cycle. The solid line represents the log hazards ratio, and the shaded area is the 95% confidence interval. ARDI = average relative dose intensity.

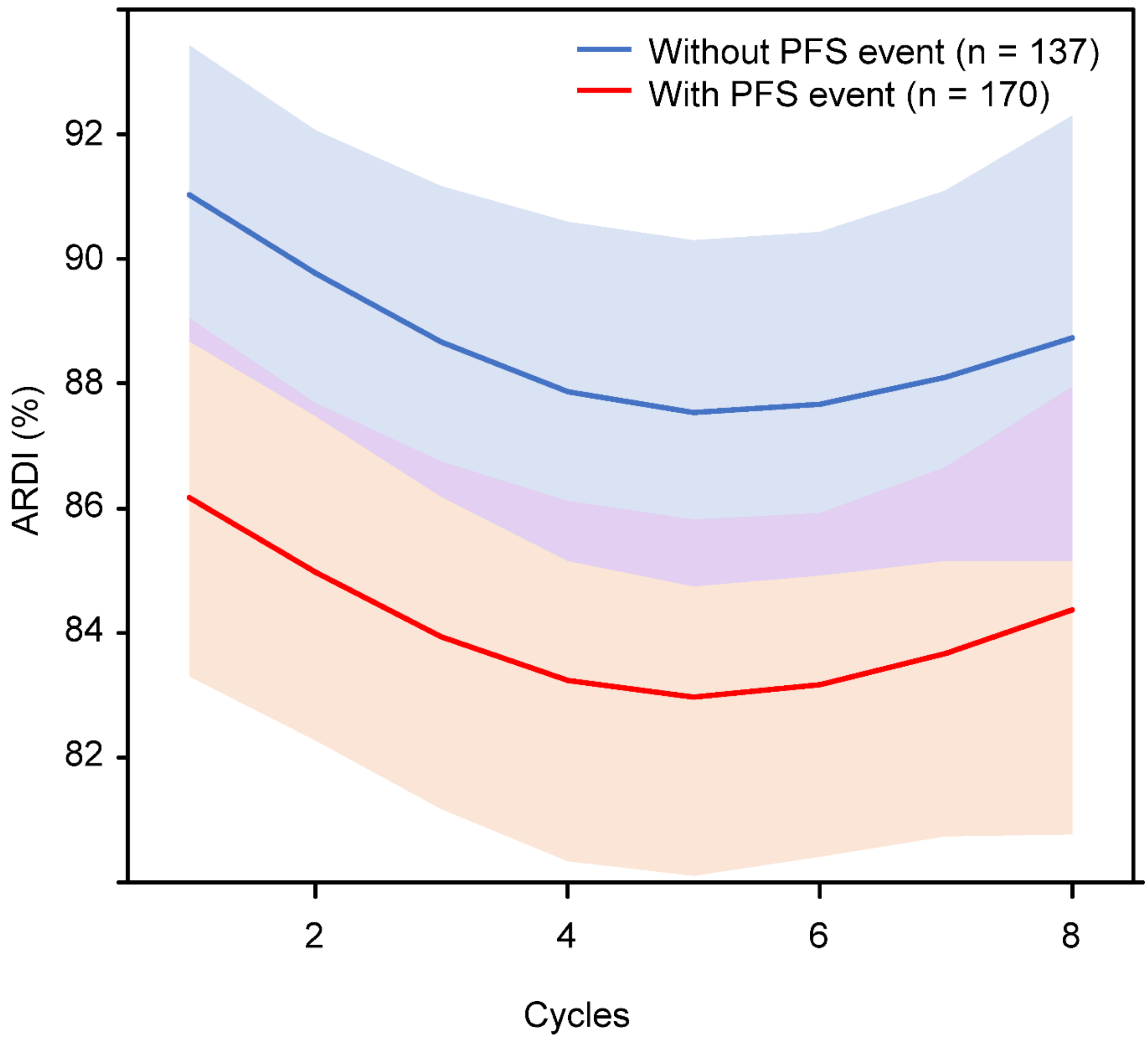

Supplementary Figure S3. Association between average relative dose intensity during each chemotherapy cycle and progression-free survival event using a multivariate Cox hazards model with restricted cubic spline with 3 knots in each of the survival and deceased patient groups. The solid line represents the log hazards ratio, and the shaded area shows the 95% confidence interval. ARDI = average relative dose intensity.

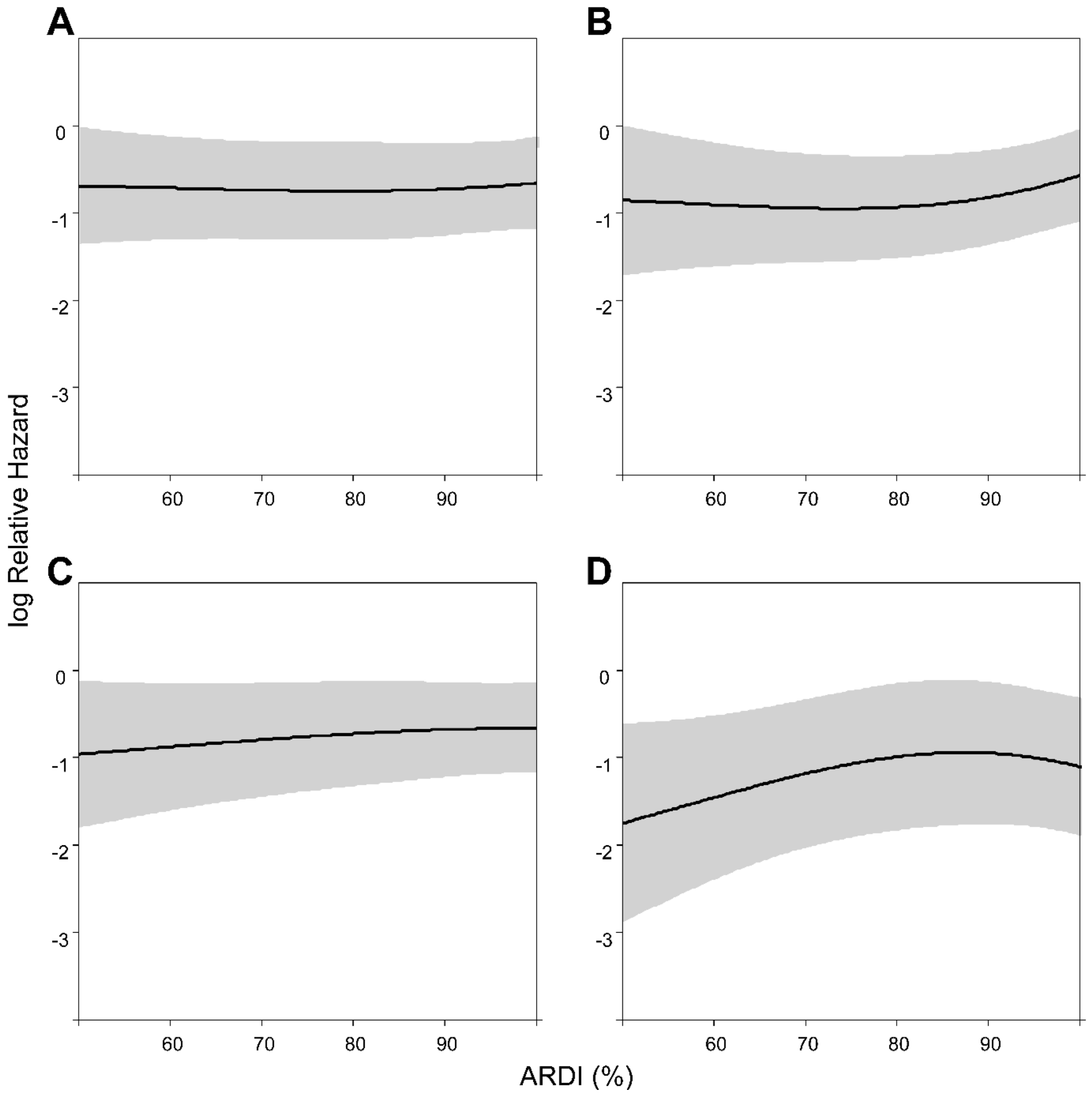

Supplementary Figure S4. Association between average relative dose intensity and progression-free survival event using a multivariate Cox hazards model with restricted cubic spline with 3 knots. (**A**) Up to the second cycle. (**B**) Up to the fourth cycle. (**C**) Up to the sixth cycle. (**D**) Up to the eighth cycle. The solid line represents the log hazards ratio, and the shaded area is the 95% confidence interval. ARDI = average relative dose intensity.
